# Association between Nonfood Pre- or Probiotic Use and Cognitive Function: Results from NHANES 2011–2014

**DOI:** 10.3390/nu15153408

**Published:** 2023-07-31

**Authors:** Jingyi Chen, Nian Yang, Yilei Peng, Honghao Zhou, Qing Li

**Affiliations:** 1 Institute of Precision Medicine, The First Affiliated Hospital of Shantou University Medical College, Shantou 515041, China; 19jychen@stu.edu.cn (J.C.); 20nyang@stu.edu.cn (N.Y.); 2 Department of Clinical Pharmacology, Xiangya Hospital, Central South University, Changsha 410008, China; 228101062@csu.edu.cn; 3 Institute of Clinical Pharmacology, Central South University, Hunan Key Laboratory of Pharmacogenetics, Changsha 410078, China

**Keywords:** probiotic, prebiotic, cognitive function, NHANES

## Abstract

In this study, we collected data from the National Health and Nutrition Examination Survey (NHANES) for the years 2011–2014. Multiple linear regression and logistic regression were used to analyse the association between nonfood pro- or prebiotic use and cognitive function among elderly Americans. To estimate the potential unobserved results, propensity score matching (PSM) was used to analyse the causal effect. Nonfood pro- or prebiotic use was analysed through the Dietary Supplement Use 30-Day Study. Cognitive function was evaluated by the Digit Symbol Substitution Test (DSST), the Animal Fluency Test (AFT), the Consortium to Establish a Registry for Alzheimer’s Disease (CERAD), and a composite Z-score calculated by summing the Z-scores of three tests. Male participants who used nonfood pro- or prebiotics tended to have higher comprehensive cognitive function (sum.z) with a β-coefficient of 0.64 (95% CI: 0.08–1.19). Probiotics or prebiotics may be a protective factor against cognitive impairment in males, with an odds ratio of 0.08 (95% CI: 0.02–0.29). Furthermore, the average treatment effect for the treated (ATT) with nonfood pro- or prebiotics (0.555) on sum.z in males was statistically significant (*p* < 0.05). Our research revealed that nonfood pre- or probiotic use was an effective method to improve cognitive function in elderly men from the USA.

## 1. Introduction

Aging can have negative effects on cognitive skills, including learning and memory. Cognitive health has emerged as a critical public health concern, especially for the elderly. The elderly are expected to account for more than one-fifth of the world’s population by 2050 [1]. In the United States, the population aged 65 and above is expected to nearly double from 52 million in 2018 to 95 million in 2060. This increase will lead to a higher proportion of this age group in the total population, rising from 16% to 23% [2]. Therefore, the number of Americans suffering from age-related cognitive decline is expected to increase [3]. This decline may be caused by a combination of genetic and environmental factors, as well as physiological, psychological, social, lifestyle, and dietary considerations [4].

There are multiple causes for cognitive impairment, but one potential factor is that the decrease of microbial diversity in elder individuals results in disruption of intestinal barrier permeability [5]. Recent studies have revealed that gut microbiota can affect brain function and behaviour. The gut–brain axis not only maintains the muscular, sensory, and secretory pathways in the gastrointestinal tract but also affects brain growth, function, and behaviour [6]. The gut microbiota plays a circular role in microglia functions, neuronal shape, and blood–brain barrier integrity [7]. Compared with young people, elderly people have a lower abundance of beneficial microbiota, specifically *Bifidobacterium* and *Lactobacillus* [8]. Probiotics are food ingredients or supplements that contain living microbes, while prebiotics are composed of non-digestive substrates which selectively stimulate the growth of beneficial microbes. The intake of prebiotics can elevate the levels of beneficial gut microbiota in older adults [9]. In addition, probiotic supplements can suppress the NF-κB signalling pathway mediated by TLR4 and RIG-I, as well as the inflammatory response, thereby improving cognitive function in aged SAMP8 mice [10]. Therefore, the intake of probiotics or prebiotics may have positive effects on human health. Moreover, the consumption of pre- or probiotics is high in the United States, with the highest intake found among older adults, reaching 8.8% [11]. Based on these findings, there is a growing interest in using nonfood pre- or probiotics as medicine to regulate the gut microbiota and return to a more physiological state.

Although some systematic reviews on the effects of pre- or probiotics on cognitive outcomes have been performed, no consistent conclusions have been drawn [12,13], which are insufficient to provide definitive evidence that the use of pre- or probiotics has effects on cognitive function. Therefore, we aimed to conduct a well-controlled and population-based study to better understand the role of nonfood pre- or probiotics in cognitive function. In this study, we aimed to investigate the association between nonfood pre- or probiotic use and cognitive function in older adults through analysing the data from the National Health and Nutrition Examination Survey (NHANES) for the years 2011–2014.

## 2. Materials and Methods

### 2.1. Population under Investigation

**Figure 1:**
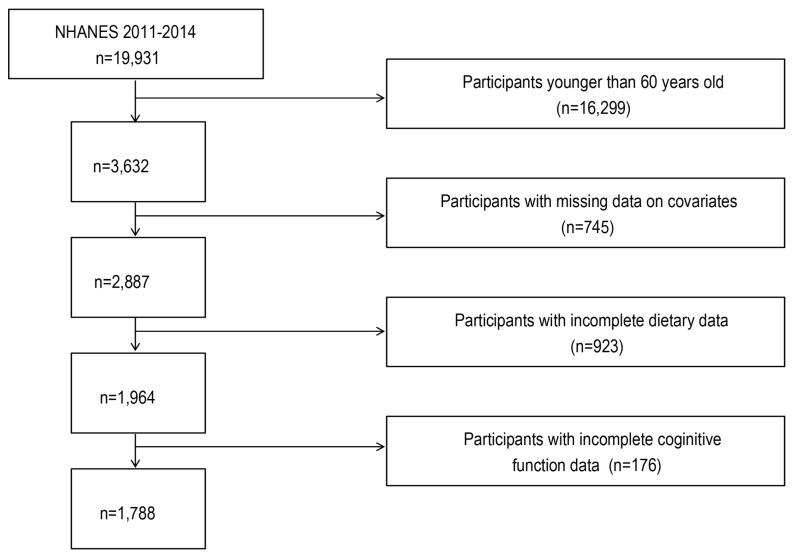
The flow diagram of the sample screening procedure.

NHANES is a study adopting a multistage sampling approach to assess health conditions and lifestyle alterations in the United States. These data are collected through personal interviews, physical assessments, biological specimen collection, and field investigations involving representative samples from the national population. We collected data on 19,931 individuals from the NHANES (2011–2014). Then, we excluded the interviewees under 60 years old (n = 16,299) and those with missing data on BMI, smoking, drinking, hypertension, stroke, diabetes mellitus (DM) and cardiovascular disease (CVD), and stroke (n = 745). Additionally, participants who lacked pre- or probiotic dietary supplement information (n = 923) and those who did not receive cognitive function tests or failed to complete four cognitive tests were also excluded (n = 176). Finally, only 1788 participants were included in our analysis, as shown in Figure 1, which describes the whole screening procedure.

### 2.2. Assessment of Nonfood Pre- or Probiotic Use

We analysed the Dietary Supplement Use 30-Day Study before the interview date to determine whether the sample used nonfood pre- or probiotics. Detailed nonfood pre- or probiotic information can be found in Table S1 [11].

### 2.3. Cognitive Functioning Evaluation

The NHANES cognitive functioning test was conducted at the Mobile Exam Center (MEC), which consisted of the CERAD word learning test (CERAD), Animal Fluency Test (AFT) and Digit Symbol Substitution Test (DSST) (https://wwwn.cdc.gov/Nchs/Nhanes/2013-2014/CFQ_H.htm (accessed on March 2017)). We used Z-score to standardise the scores of CERAD, AFT, and DSST. The sum of the three standardised scores is recorded as ‘sum.z’.

### 2.4. Covariates

The NHANES collects information on demographic, socioeconomic, and health-related issues. We adopted some of them as covariates, including age (60–70 years and ≥70 years), gender (male and female), ethnicity (Mexican-American, non-Hispanic white, non-Hispanic black, and others), educational level (less than high school, high school or higher) [14], ratio of family income to poverty (PIR) (<1.3, ≥1.3–3.5, and >3.5), body mass index (BMI) (normal: <25 kg/m^2^, overweight: 25 to <30 kg/m^2^, obesity: ≥30 kg/m^2^) [15], drinking (never, former, current) [16] and smoking status (never, former, current) [17]. In addition, disease history (hypertension, stroke, DM, and CVD) was included as covariates.

### 2.5. Statistical Analysis

We chose ‘wtmec2yr’ for 2011–2014 and calculated these weights using the following formula:

wt = 1/2 ∗ WTDR2D.(1)

Continuous data were represented by mean and standard deviation (SD), while categorical variables were denoted by sample size and weighted percentage (%). The participants were divided into two groups: one group included individuals who used nonfood pre- or probiotics, and the other group comprised those who did not consume either. To investigate the relationship between cognitive function and nonfood pre- or probiotic use, we conducted a linear regression analysis with defined risk factors of cognitive function as covariates based on previous studies [18]. Model 1 did not make any adjustments. Model 2 adjusted for age, gender, race, educational level, PIR, BMI, alcohol drinking status, and smoking status. Model 3 further adjusted for hypertension, stroke, DM, and CVD in addition to the factors from Model 2. We also stratified our analysis by gender, age, ethnicity, and BMI to evaluate their impact on cognitive function. Furthermore, we analysed the interaction effects of these three factors (age, ethnicity, and BMI) with nonfood pre- or probiotic use. The *p* value < 0.05 was statistically significant.

To better understand the effects of nonfood pre- or probiotic use on cognitive impairment, we performed a logistic regression analysis. Since there is no clear diagnostic measure for cognitive impairment, we established the cutoff point for the two age groups based on previous studies to define cognitive impairment [19]. The lowest quartile of sum.z was used as the threshold, with −0.812 for 60–69 years old and −2.311 for ≥70 years old. All analyses were performed using R (4.2.2) software.

Although regression models have been used to investigate the relationship between nonfood pre- or probiotic use and cognitive function, cross-sectional observational research is methodologically challenged by the limitation of causal inference. In order to address this limitation when randomized data are unattainable, we adopted propensity score matching (PSM) as a suitable alternative for estimating effects. Using binary random variables ‘Di’ to represent whether nonfood pre- or probiotics were used or not and ‘Yi’ to measure comprehensive cognitive function (sum.z), we established a simple linear regression model:

Yi = α + βDi + μi,(2)

The value of i = 1 represents ‘nonfood pre- or probiotic use’, while the value of i = 0 represents ‘no nonfood pre- or probiotic use’. Kernel matching was adopted to eliminate bias, and the average treatment effect for the treated (ATT), representing the β-coefficient, was denoted as the anticipated difference in outcomes between the nonfood pre- or probiotic use group and the group with no nonfood pre- or probiotic use [20]. The data analysis was performed using Stata (15.1) software.

## 3. Results

### 3.1. Descriptive Statistics

This analysis included 1788 elderly individuals aged 60 or above. In this study cohort, the average age of the two groups was 69.05 for those who used nonfood pre- or probiotics and 69.36 for those who did not. Participants who used nonfood pre- or probiotics were more likely to be white. The baseline characteristics of the population are displayed in Table 1. Three different cognitive tests (z.CERD, z.AFT, and z.DSST) and their sum (sum.z) were compared between the participants who used nonfood pre- or probiotics and those who did not. The *p*-values indicated statistical significance between the two groups for each test. The sum.z, which represents the comprehensive cognitive function, showed an improvement of 0.68 when comparing the participants who used nonfood pre- or probiotics with those who did not.

Furthermore, we compared cognitive function by gender among the participants who did not use nonfood pre- or probiotics. The results revealed that females had worse comprehensive cognitive function than males (0.54 vs. 0.91) in the group that did not use nonfood pre- or probiotics (Table S2).

### 3.2. Modulation of Cognitive Function Score According to Nonfood Pre- or Probiotic Use

To explore the relationship between cognitive function and nonfood pre- or probiotic use, we performed a multiple linear regression analysis with a crude model and two multivariable-adjusted models (Table 2). In the crude model (Model 1), the better performance of cognitive function was significantly associated with the use of nonfood pre- or probiotics. The β-coefficient for z.DSST and comprehensive cognitive function (sum.z) were 0.29 (95% CI: 0.11–0.47) and 0.67 (95% CI: 0.14–1.21), respectively. However, after adjusting for age, sex, race, BMI, smoking, alcohol use, education, and poverty (Model 2) and further adjusting for diseases including hypertension, stroke, DM, and CVD (Model 3), no significant relationship was found. Furthermore, we carried out a subgroup analysis and found that males performed better cognitive functions. In Model 1, the β-coefficient of z.DSST was 0.41 (95% CI: 0.11,0.72) and the β-coefficient of sum.z was 1.09 (95% CI: 0.30,1.88). After adjusting for covariates, the association between sum.z and nonfood pre- or probiotic use remained significant, with the value of 0.73 (95% CI: 0.19 to 1.27) in Model 2 and 0.64 (95% CI: 0.13 to 1.24) in Model 3, although the coefficients were attenuated (Table 2). However, no significant association was found in females.

### 3.3. Interaction Effects

**Figure 2:**
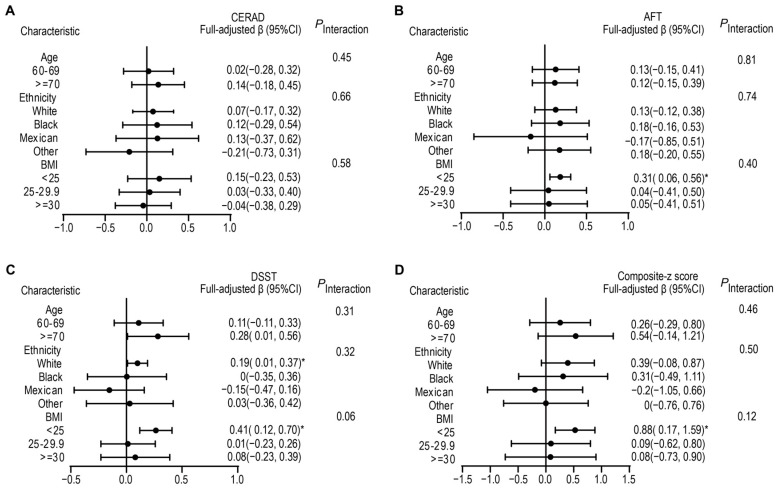
Association between nonfood pre- or probiotic use and different cognitive function scores stratified by age, ethnicity, and BMI. Adjusted for age, gender, ethnicity, BMI, drink, smoke, education, PIR, hypertension, stroke, DM, and CVD. The strata variable was not included in the model when stratifying by itself. (**A**) CERAD; (**B**) AFT; (**C**) DSST; (**D**) Composite-z score (sum.z). * *p* < 0.05.

To learn more about the interaction between nonfood pre- or probiotic use and factors such as age, ethnicity, and BMI, we conducted interaction analyses on these variables. The results indicated that there was no interaction between these variables in the model when other covariates were considered. However, participants who used nonfood pre- or probiotics showed better z.AFT, z.DSST, and sum.z in the population of BMI < 25, with β-coefficients of 0.31(95% CI: 0.06, 0.56), 0.41 (95% CI: 0.12, 0.70) and 0.88 (95% CI: 0.17, 1.59), respectively (Figure 2). Moreover, white individuals who used pre -or probiotics showed better z.DSST, with the β-coefficient of 0.19 (95% CI: 0.01, 0.37).

### 3.4. Cognitive Impairment and Nonfood Pre- or Probiotic Use

To further explore the association between nonfood pre- or probiotic use and cognitive impairment, the sum.z score was divided into quartiles based on age subgroups, with the lowest quartile considering cognitive impairment. The basic characteristics of the two groups are displayed in Table 3. The results showed that participants with cognitive impairment used almost none of the nonfood pre- or probiotics. Subsequently, a logistic regression analysis was conducted to examine the association between nonfood pre- or probiotic use and cognitive impairment in males and females. The results suggested that nonfood pre- or probiotic use was a protective factor for cognition impairment in males, with significant odds ratios in both the unadjusted model and the two adjusted models (*p* < 0.001), while no significant difference was observed in women in the adjusted models (Table 4).

### 3.5. Balance Test and PSM Results

To estimate the causal effect of nonfood pre- or probiotic use on the sum.z score, we used propensity score matching (PSM) to avoid selectivity bias caused by potential outcomes. As previous analyses have indicated that there is gender bimodality in the impact of nonfood pre- or probiotic use on cognitive function, we stratified our samples by gender and conducted PSM on males and females separately. Before kernel matching, we redefined several covariates, including age, BMI, PIR, education level, smoking status, alcohol consumption, hypertension, stroke, DM, and CVD, as shown in Table S3. Table 5 and Table 6 show the balance tests for males and females. The results showed that after matching, all covariates between the nonfood pre- or probiotic use group and the no nonfood pre- or probiotic use group were almost balanced (*p* < 0.05), which meant that sample equilibrium was achieved to some extent. Table 7 shows the ATT for males and females. For males, the result was significantly different (*p* < 0.05) between the treated group and the control group, with a difference of 0.555 and a standard error (SE) of 0.282. However, for females, the difference was not statistically significant (*p* > 0.05), with a difference of 0.235 and a standard error of 0.266. From this, it is suggested that nonfood pre- or probiotic use is an effective method to improve cognitive function in elderly men.

## 4. Discussion

This cross-sectional analysis aimed to evaluate the association between nonfood pre- or probiotic use and cognitive function in older adults. We found that nonfood pre- or probiotic use is significantly positively correlated with comprehensive composite cognitive function, particularly among males, both before and after adjusting for demographic and potential confounding factors. Moreover, obesity significantly altered the association between nonfood pre- or probiotic use and cognitive function. Our results suggest that alterations in the gut microbiota may contribute to the prevention of cognitive impairment in older adults. In previous studies, the association between nonfood pre- or probiotic use and cognitive function has been inconsistent. Several meta-analyses have shown that probiotic treatment improves cognitive impairment [21,22,23,24]. However, some studies do not support the positive effects of probiotics, prebiotics, and fermented foods on cognitive function in elder populations [12,25,26]. Inconsistent results may be due to different sociodemographic characteristics or small sample sizes. In our analysis, we found that nonfood pre- or probiotic use improved comprehensive cognitive function. This study is a national survey with a large sample size, providing reliable evidence. Furthermore, we conducted subgroup analyses by examining demographic characteristics (gender, age, ethnicity, and BMI). Males and individuals with a BMI < 25 are more likely to benefit from nonfood pre- or probiotic use. It is well-known that elder women with higher oestrogen levels are at higher risk for Alzheimer’s disease. The observed gender dimorphism in cognitive function may be due to the metabolism of sex hormones regulated by the gut microbiota [27]. Therefore, preventive strategies for cognitive impairment in women may need to consider other intervention measures. Some evidence also suggests that obesity can impair cognitive function [28,29], leading us to speculate that obesity may interfere with the effectiveness of probiotics or prebiotics. Obesity is a metabolic disorder and is associated with gut microbiota dysbiosis, which may affect the function of prebiotics and probiotics in the gut. Therefore, it may be necessary to consider the relationship between obesity, gut microbiota, and cognitive function when designing interventions for improving cognitive function strategies with prebiotics or probiotics. In the future, research focusing on the effects of specific probiotic and prebiotic types on individuals with different BMI levels may develop more personalized intervention strategies to meet the needs of different populations, thereby improving the success rate and applicability of these approaches. In addition, it is important to explore how obesity interferes with the effectiveness of prebiotics or probiotics.

To further understand the effects of nonfood pre- or probiotic use on cognitive function, after conducting correlational analyses (multiple linear regression and multiple logistic regression), we also adopted the kernel matching method for propensity scores to infer causal relationships between nonfood pre- or probiotic use and cognitive function. PSM is primarily used to address confounding biases in estimating causal effects in observational studies. By matching individuals with similar propensity scores in the experimental and control groups, the reliability of causal inference is improved by simulating a randomized controlled trial. ATT was calculated to assess the effect of nonfood pre- or probiotic use on cognitive function. Our results indicated (ATT = 0.555, *p* < 0.05) that, after considering confounding factors, nonfood pre- or probiotic use had a significant positive effect on cognitive function in males. However, it should be noted that this study was based on observational data. Although propensity score matching (PSM) was used to control for potential confounding factors, there may still be some unconsidered confounders, which may continue to bias results. Therefore, it just implies that nonfood pre- or probiotic use is a potential causal relationship and could be an effective strategy for improving cognitive function in elderly males.

This study has several advantages. First, it avoids the heterogeneity of small samples by using a large, nationally representative sample. Second, cognitive function was assessed by using three cognitive scores (DSST, AFT, and CEARD) and a composite cognitive score (sum.z). Multiple linear regression was used to analyse the effect of nonfood pro- or prebiotic use on cognitive function, and then logistic regression was used to further confirm that nonfood pro- or prebiotic use was a protective factor for cognitive impairment. In the regression analyses, covariates were also adjusted to eliminate potential confounding effects. Third, this study was an observational study, so causal inference was methodologically challenging. To address this limitation, and to perform causal inference when random data are not available, we used kernel matching to eliminate biases and propensity score matching (PSM) in the form of ATT to interpret potential causal effects. However, there are some limitations to this study. First, dietary data were collected in the NHANES questionnaire, which may introduce information bias. For instance, data from the Dietary Supplement Use 30-Day Study may be subject to recall bias. Second, in this study, we only analysed whether the participants were using nonfood pro- or prebiotics. Since it is difficult to standardize the amount or unit of pre- or probiotics in the diet, we did not quantify their intake, so we were unable to explore the effect of different types or amounts of pre- or prebiotics on cognitive function. Moreover, the data from the Dietary Supplement Use 30-Day Study only recorded participants’ intake, not the duration of use of pre- or prebiotic supplements. Therefore, it is difficult to distinguish between short-term and long-term differences. Despite some limitations, the obtained results have been encouraging and motivating for further research in this direction. Further long-term prospective research is necessary.

## 5. Conclusions

In conclusion, by adopting a nationally representative cohort of elder people from the USA, we found that the positively association between nonfood pre- or probiotic use and cognitive function is stronger in males, the population of BMI < 25 and white individuals. Our study emphasized that nonfood pre- or probiotic use is an effective method to improve cognitive function in elderly men.

## Figures and Tables

**Table 1:** Characteristics of all participants according to nonfood pre- or probiotic use.

Characteristic	Nonfood Pre- or Probiotic Use	No Nonfood Pre- or Probiotic Use	*p*
	135 (7.56%)	1653 (92.4%)	
Age	69.05 (67.78, 70.32)	69.36 (68.92, 69.80)	0.65
Age_subgroup			0.72
60–69	67 (57.00)	838 (54.86)	
≥70	68 (43.00)	815 (45.14)	
Gender			0.56
Female	68 (54.94)	940 (57.55)	
Male	67 (45.06)	713 (42.45)	
Ethnicity			0.01
White	87 (89.63)	892 (82.67)	
Black	26 (4.81)	345 (6.95)	
Mexican	8 (1.85)	120 (2.63)	
Other	14 (3.71)	296 (7.75)	
Education			0.1
Less than high school	12 (6.51)	347 (13.31)	
High school or higher	123 (93.49)	1306 (86.69)	
PIR			0.16
<1.3	21 (9.34)	448 (15.62)	
1.3–3.5	53 (37.32)	632 (37.96)	
>3.5	61 (53.34)	573 (46.42)	
BMI			0.32
<25	40 (32.50)	444 (25.91)	
25–29.9	45 (37.06)	588 (36.82)	
≥30	50 (30.44)	621 (37.27)	
Smoker			0.75
Never	68 (52.96)	855 (51.63)	
Former	57 (40.19)	617 (39.00)	
Current	10 (6.84)	181 (9.37)	
Alcohol			0.37
Current	82 (69.56)	928 (64.13)	
Former	38 (21.66)	458 (22.57)	
Never	15 (8.78)	267 (13.30)	
Hypertension			0.12
No	45 (41.40)	470 (32.25)	
Yes	90 (58.60)	1183 (67.75)	
Stroke			0.28
No	123 (90.64)	1534 (93.70)	
Yes	12 (9.36)	119 (6.30)	
DM			0.43
No	85 (66.90)	958 (63.43)	
Yes	50 (33.10)	695 (36.57)	
CVD			0.57
No	103 (76.22)	1283 (78.78)	
Yes	32 (23.78)	370 (21.22)	
z.CERD	0.27 (0.03, 0.51)	0.14 (0.05, 0.24)	0.3
z.AFT	0.51 (0.26, 0.75)	0.26 (0.18, 0.33)	0.05
z.DSST	0.61 (0.45, 0.76)	0.31 (0.25, 0.38)	0.002
sum.z	1.39 (0.88, 1.89)	0.71 (0.53, 0.90)	0.02

PIR: ratio of family income to poverty; BMI: body mass index; DM: diabetes mellitus; CVD: cardiovascular disease.

**Table 2:** Regression coefficients and 95% confidence intervals in nonfood pre- or probiotic use group when compared with no nonfood pre- or probiotic use group.

	z.AFT	z.CEART	z.DSST	Sum.z
All participants (n = 1788)				
Model 1	0.25 (0.00, 0.50)	0.13 (−0.12, 0.38)	0.29 (0.11, 0.47) **	0.67 (0.14, 1.21) *
Model 2	0.14 (−0.08, 0.36)	0.06 (−0.17, 0.29)	0.15 (−0.01, 0.32)	0.35 (−0.08, 0.78)
Model 3	0.13 (−0.09, 0.36)	0.07 (−0.17, 0.30)	0.16 (−0.01, 0.33)	0.36 (−0.09, 0.80)
Male (n = 780)				
Model 1	0.4 (−0.02, 0.83)	0.27 (−0.05, 0.60)	0.41 (0.11, 0.72) *	1.09 (0.30, 1.88) *
Model 2	0.29 (−0.07, 0.65)	0.19 (−0.07, 0.45)	0.25 (0.00, 0.50)	0.73 (0.19, 1.27) *
Model 3	0.25 (−0.11, 0.61)	0.18 (−0.09, 0.45)	0.26 (−0.01, 0.52)	0.69 (0.13, 1.24) *
Female (n = 1008)				
Model 1	0.12(−0.15, 0.39)	0.03 (−0.31, 0.37)	0.2 (−0.01, 0.41)	0.35 (−0.35, 1.05)
Model 2	0.01 (−0.25, 0.27)	−0.05 (−0.36, 0.25)	0.06 (−0.13, 0.25)	0.01 (−0.57, 0.60)
Model 3	0.03 (−0.24, 0.31)	−0.03 (−0.35, 0.29)	0.08 (−0.12, 0.29)	0.09 (−0.54, 0.72)

Model 1: no adjustment; Model 2: adjusted for age, gender, ethnicity, educational level, PIR, BMI, smoke, and drink; Model 3: adjusted for all the factors in Model 2 plus hypertension, stroke, DM, and CVD. PIR: ratio of family income to poverty; BMI: body mass index; CVD: cardiovascular disease. * *p* < 0.05, ** *p* < 0.01

**Table 3:** Characteristics of all participants in cognitive impairment and non-cognitive impairment groups.

Characteristic	Cognitive Impairment	Non-Cognitive Impairment	*p*
	447 (25.00)	1341 (75.00)	
Age	71.51 (70.70, 72.33)	68.94 (68.46, 69.42)	<0.0001
Age_subgroup			0.003
60–69	226 (46.49)	679 (56.59)	
≥70	221 (53.51)	662 (43.41)	
Gender			0.57
Female	223 (55.54)	785 (57.64)	
Male	224 (44.46)	556 (42.36)	
Ethnicity			<0.0001
White	157 (65.82)	822 (86.43)	
Black	143 (16.20)	228 (5.06)	
Mexican	48 (6.15)	80 (1.92)	
Other	99 (11.83)	211 (6.59)	
Education			<0.0001
Less than high school	180 (29.57)	179 (9.67)	
High school or higher	267 (70.43)	1162 (90.33)	
PIR			<0.0001
<1.3	205 (36.65)	264 (11.18)	
1.3–3.5	151 (40.26)	534 (37.48)	
>3.5	91 (23.09)	543 (51.34)	
BMI			0.59
<25	122 (29.01)	362 (26.05)	
25–29.9	157 (35.00)	476 (37.17)	
≥30	168 (35.98)	503 (36.78)	
Smoker			0.02
Never	229 (52.43)	694 (51.63)	
Former	151 (34.63)	523 (39.91)	
Current	67 (12.94)	124 (8.46)	
Alcohol			<0.0001
Current	183 (43.27)	827 (68.45)	
Former	163 (32.51)	333 (20.69)	
Never	101 (24.22)	181 (10.86)	
Hypertension			<0.0001
No	101 (19.10)	414 (35.58)	
Yes	346 (80.90)	927 (64.42)	
Stroke			0.003
No	388 (86.10)	1269 (94.74)	
Yes	59 (13.90)	72 (5.26)	
DM			0.01
No	230 (54.74)	813 (65.35)	
Yes	217 (45.26)	528 (34.65)	
CVD			0.01
No	310 (68.38)	1076 (80.37)	
Yes	137 (31.62)	265 (19.63)	
Group			<0.001
No pre- or probiotic use	432 (97.29)	1221 (89.89)	
Pre- or probiotic use	15 (2.71)	120 (10.11)	

PIR: ratio of family income to poverty; BMI: body mass index; CVD: cardiovascular disease.

**Table 4:** Odds ratio (95% confidence intervals) of nonfood pre- or probiotic use in cognitive impairment in males and females.

	Model 1		Model 2		Model 3	
	OR (95% CI)	*p*	OR (95% CI)	*p*	OR (95% CI)	*p*
Male	0.06 (0.02, 0.19)	<0.0001	0.08 (0.02, 0.25)	<0.001	0.08 (0.02, 0.27)	<0.001
Female	0.38 (0.17, 0.83)	0.02	0.52 (0.21, 1.26)	0.14	0.50 (0.20, 1.24)	0.13

Model 1: no adjustment; Model 2: adjusted for age, gender, ethnicity, educational level, PIR, BMI, smoke, and drink; Model 3: adjusted for all the factors in Model 2 plus hypertension, stroke, diabetes, and CVD; PIR: ratio of family income to poverty; BMI: body mass index; DM: diabetes mellitus; CVD: cardiovascular disease.

**Table 5:** Balance test of covariable in males.

Variable	Sample	Mean Value		Standard Bias (%)	Bias Reduction (%)	T	*p*
		Nonfood Pre- or Probiotic Use	No Nonfood Pre- or Probiotic Use				
Age	U	70.015	70.289	−4.1		−0.32	0.752
	M	70.015	70.212	−2.9	28.2	−0.17	0.867
Ethnicity	U	0.731	0.889	−14.5		−1.09	0.277
	M	0.731	0.792	−5.6	61.7	−0.32	0.746
PIR	U	1.478	1.149	45.3		3.32	0.001
	M	1.478	1.377	13.9	69.4	0.84	0.401
Education	U	0.94	0.781	47.1		3.1	0.002
	M	0.94	0.927	4	91.5	0.31	0.756
BMI	U	1.03	1.077	−6.1		−0.48	0.628
	M	1.03	1.059	−3.8	37.6	−0.22	0.827
Smoking	U	0.642	0.776	−21.1		−1.58	0.114
	M	0.642	0.7	−9.3	56.2	−0.54	0.589
Alcohol	U	1.642	1.539	16.8		1.28	0.201
	M	1.642	1.594	7.7	54.1	0.45	0.654
Hypertension	U	0.612	0.697	−17.9		−1.44	0.15
	M	0.612	0.663	−10.7	40.4	−0.61	0.545
Stroke	U	0.06	0.067	−3.1		−0.24	0.811
	M	0.06	0.061	−0.6	80.5	−0.04	0.971
CVD	U	0.224	0.264	−9.2		−0.71	0.478
	M	0.224	0.242	−4.2	54.3	−0.25	0.805
DM	U	0.358	0.467	−22.2		−1.71	0.088
	M	0.358	0.406	−9.7	56.1	−0.57	0.573

PIR: ratio of family income to poverty; BMI: body mass index; DM: diabetes mellitus; CVD: cardiovascular disease; M: matched; U: Unmatched.

**Table 6:** Balance test of covariable in females.

Variable	Sample	Mean Value		Standard Bias (%)	Bias Reduction (%)	T	*p*
		Nonfood Pre- or Probiotic Use	No Nonfood Pre- or Probiotic Use				
Age	U	70.235	69.766	7.1		0.55	0.584
	M	70.235	70.013	3.4	52.6	0.19	0.846
Ethnicity	U	0.515	0.893	−35.7		−2.64	0.008
	M	0.515	0.67	−14.7	59	−0.91	0.362
PIR	U	1.118	1.02	12.8		1	0.316
	M	1.118	1.072	6	53.5	0.35	0.728
Education	U	0.882	0.797	23.4		1.71	0.087
	M	0.882	0.846	10	57.2	0.62	0.537
BMI	U	1.118	1.13	−1.5		−0.12	0.906
	M	1.118	1.133	−1.9	−28.3	−0.11	0.913
Smoking	U	0.5	0.453	7.1		0.57	0.569
	M	0.5	0.473	4.1	42.4	0.24	0.812
Alcohol	U	1.353	1.295	7.5		−0.16	0.869
	M	1.353	1.325	3.5	52.9	−0.05	0.962
Hypertension	U	0.721	0.73	−2.1		1.25	0.212
	M	0.721	0.724	−0.8	59.8	0.83	0.408
Stroke	U	0.118	0.076	14.2		1.13	0.26
	M	0.118	0.075	14.3	−0.3	0.72	0.474
CVD	U	0.25	0.194	13.5		−0.05	0.964
	M	0.25	0.198	12.4	8.3	0.04	0.971
DM	U	0.382	0.385	−0.6		0.57	0.566
	M	0.382	0.379	0.6	−12.4	0.21	0.838

PIR: ratio of family income to poverty; BMI: body mass index; DM: diabetes mellitus; CVD: cardiovascular disease; M: matched; U: Unmatched.

**Table 7:** PSM result in males and females.

	Nonfood Pre- or Probiotic Use	No Nonfood Pre- or Probiotic Use	Difference (ATT)	SE	T	*p*
Male	0.54	−0.015	0.555	0.282	1.97	<0.05
Female	0.604	0.37	0.235	0.266	0.88	>0.05

ATT: the average treatment effect for the nonfood pre- or probiotic use.

## Data Availability

Not applicable.

## Supplementary Materials

The following supporting information can be downloaded at: https://www.mdpi.com/article/10.3390/nu15153408/s1. Table S1: The classification of probiotics and prebiotics; Table S2: Charateristics of participants who did not use pre- or probiotic; Table S3: Definition various variables for PSM.
