# Supplementary material for: Association between Nonfood Pre- or Probiotic Use and Cognitive Function: Results from NHANES 2011–2014
Source: Nutrients. 2023 Jul 31;15(15):3408. doi: 10.3390/nu15153408 (PMC10421231; doi:10.3390/nu15153408)
Supplement: Supplementary file 1 [file nutrients-15-03408-s001.zip › nutrients-2532140-supplementary.pdf]

Table S1. The classification of probiotics and prebiotics.

**Prebiotic**	**Probiotic**
glucan, gum, arabic, inulin, oligofruc, oligosac, prebiotic, pre-biotic, resistant starch, chicor, psyllium, resveratrol, lactulose	acidophilus, animalis, bacillus, bacilli, bifidobacteri, bifidum, boulardii, breve, brevis, bulgaricus, casei, cerevisiae, coagulans, delbrueckii, enterococcus, faecalis, faecium, fermentum, gasseri, helveticus, infantis, lactis, lactic acid bacteria, lactobacill, lactococcus, leuconostoc, licheniformis, longum, mesenteric, paracasei, pediococcus, plantarum, probiotic, pro-biotic, pro biotic, propionibacteri, reuteri, rhamnosus, saccharomyc, salivarius, streptococcus, subtilis, thermophilus, E. coli

Table S2. Charateristics of participants who did not use nonfood pre- or probiotic.

Characteristic	Female	Male	*P*
Age	69.56(69.06,70.05)	69.09(68.42,69.76)	0.23
Age_subgroup			0.24
60-69	484(53.69)	356(56.72)	
>=70	452(46.31)	361(43.28)	
Ethnicity			0.24
White	516(82.40)	389(84.25)	
Black	187(7.38)	147(5.48)	
Mexican	64(2.62)	58(2.72)	
Other	169(7.60)	123(7.55)	
Education			0.73
Less than high school	184(12.70)	149(12.10)	
High school or higher	752(87.30)	568(87.90)	
PIR			< 0.0001
<1.3	275(18.78)	170(11.35)	
1.3-3.5	371(39.76)	254(33.86)	
>3.5	290(41.46)	293(54.78)	
BMI			0.04
<25	262(29.00)	185(22.20)	
25-29.9	289(32.76)	291(41.15)	
>=30	385(38.24)	241(36.64)	
Smoke			< 0.0001
Never	593(59.76)	260(40.02)	
Former	261(31.74)	363(49.29)	
Current	82(8.50)	94(10.69)	
Alcohol			< 0.001
Current	484(61.36)	445(69.15)	
Former	242(21.42)	218(23.62)	
Never	210(17.22)	54(7.23)	
Hypertension			0.86
No	251(32.01)	218(32.45)	
Yes	685(67.99)	499(67.55)	
Stroke			0.98
No	865(93.37)	670(93.33)	
Yes	71(6.63)	47(6.67)	

DM			0.02
No	580(67.76)	391(59.44)	
Yes	356(32.24)	326(40.56)	
CVD			0.002
No	751(82.18)	527(71.97)	
Yes	185(17.82)	190(28.03)	
z.CERD	0.26(0.16,0.35)	-0.01(-0.14,0.11)	< 0.0001
z.AFT	0.23(0.14,0.33)	0.31(0.20,0.43)	0.24
z.DSST	0.42(0.34,0.50)	0.24(0.16,0.31)	< 0.001
sum.z	0.91(0.69,1.13)	0.54(0.29,0.79)	0.004

PIR: ratio of family income to poverty; BMI: body mass index; DM: diabetes mellitus; CVD: cardiovascular disease.

Table S3. Definition various variables for PSM.

Variable	Variable definitions
Treated	0=None,1=Prebiotic or probiotic
Age	Continuous variable
Ethnicity	0=white,1=black,2=mexican,3=other
PIR	0="<1.3", 1="1.3-3.5", 2=">3.5"
Education	0=Less than high school,1=High school or higher
BMI	0="<25" 1="25-29.9", 2=">30"
Smoke	0=Never,1=Former,2=Current
Alcohol	0=Never,1=Former,2=Current
Hypertension	0=No,1=Yes
Stroke	0=No,1=Yes
CVD	0=No,1=Yes
DM	0=No,1=Yes

PIR: ratio of family income to poverty; BMI: body mass index; CVD: cardiovascular disease; DM: diabetes mellitus.
